# Attitudes to suicide following the suicide of a friend or relative: a qualitative study of the views of 429 young bereaved adults in the UK

**DOI:** 10.1186/s12888-017-1560-3

**Published:** 2017-12-13

**Authors:** Alexandra Pitman, Hedvig Nesse, Nicola Morant, Valeriya Azorina, Fiona Stevenson, Michael King, David Osborn

**Affiliations:** 1 UCL Division of Psychiatry, Maple House, 149 Tottenham Court Road, London, W1T 7NF UK; 2 Camden and Islington NHS Foundation Trust, St Pancras Hospital, St Pancras Way, London, NW1 0PE UK; 3 UCL Research Department of Primary Care and Population Health, UCL Medical School, Royal Free Campus, Rowland Hill St, London NW3 2PF, UK

**Keywords:** Suicide, Self-harm, Contagion, Bereavement, Suicide prevention, Attitudes to death

## Abstract

**Background:**

People bereaved by suicide are at increased risk of suicide attempt and suicide, but explanations for these associations remain theoretical. It is possible that the experience of suicide bereavement modifies personal attitudes towards suicide, but the nature of these changes remains unexplored. There is a need to understand personal attitudes to suicide following suicide bereavement, as this may inform the development of suicide prevention interventions. Our aim was to explore the attitudes of young adults bereaved by suicide towards their own likelihood of dying by suicide.

**Methods:**

We conducted a cross-sectional study of staff and students aged 18–40 at 37 United Kingdom (UK) higher educational institutions in 2010. Ethical approval was granted by the UCL Research Ethics Committee. Qualitative responses to a question probing attitudes to own suicide were provided by 429 respondents who had experienced bereavement by the suicide of a close contact. We identified key themes in this dataset using thematic analysis.

**Results:**

Analysis identified four main themes: suicide as a more tangible option (whether feared or not); identification with the deceased and awareness of shared vulnerabilities to suicide; personal determination to avoid suicide; and beliefs regarding safeguards against suicide. These themes reflected a broad split in participants' views regarding own likelihood of dying by suicide, influenced by the degree to which own suicide was feared and the extent to which they felt in control of determining a suicide death. Whilst the majority described an aversion to the idea of attempting suicide themselves, largely through an awareness of the impact on others, a minority described their experiences as having normalised suicide as a personal option.

**Conclusions:**

The views of a sample of UK-based adults bereaved by suicide suggest that exposure to the suicide of a close friend or relative can influence attitudes to suicide in ways that could influence own risk of suicide attempt. The normalising attitudes to suicide observed in a minority of respondents could contribute to the observed association between suicide bereavement and suicide attempt.

**Electronic supplementary material:**

The online version of this article (10.1186/s12888-017-1560-3) contains supplementary material, which is available to authorized users.

## Background

For every suicide it is estimated that ten relatives or friends are deeply affected by the death [1]. These bereaved individuals have an increased risk of suicide [2] and suicide attempt [3], with one in ten people reporting having attempted suicide after the suicide of a close contact [3]. With over 800,000 people dying by suicide worldwide annually [4], evidence suggests that the past year prevalence of exposure to suicide bereavement is 4%, with a lifetime prevalence of 22% [5]. Most suicide prevention strategies highlight the need to provide support to this group [6–9] but there is weak evidence for interventions to improve their health outcomes [10] and a perceived lack of informal support [11]. A key barrier to developing effective interventions is the lack of understanding of the mechanisms of suicidality after suicide bereavement. It is critical to develop and test theories about potential explanatory factors, in order to design interventions to address modifiable risk factors. These can then be trialled for their effectiveness in reducing the risk of suicide and improving wider health outcomes for this group. Our earlier systematic review described the increased risk of suicide, psychiatric admission, and depression in people bereaved by suicide [2]. We also suggested various explanations for the association between suicide bereavement and suicide attempt, based on the literature and clinical observation [2]. These included: the psychological trauma of a suicide loss; shared familial or environmental vulnerabilities to mental illness and suicidality; the influence of stigma [12] on help-seeking; and suicide suggestion [13]. The last of these describes the impact of a role model’s suicide or suicide attempt on a person’s internal constraints against self-harm, [14] whether due to social learning, imitation or emotional contagion [13]. The phenomenon is described by a number of terms, none of which satisfactorily describe the phenomenon, but include; imitative suicide, [14] suicide contagion [15], or suicide diffusion [16]. Gaining an understanding of this phenomenon is the focus of the current study.

Personal attitudes to suicide are theorised to play a key role in forming suicidal ideas [14]. Despite the possibility of residual confounding due to shared social adversity and/or assortative homophily, [14] there is evidence to support the effect of suicide suggestion after exposure to a peer’s fatal or non-fatal suicide attempt [17, 18]. Young people are thought to be particularly susceptible to emulating suicidal behaviour of their peers [19], and this is thought to explain a number of well-publicised suicide clusters [20, 21]. There is also evidence that young people are more likely than their elders to hold accepting views towards suicide [22], regarding it as a means of expressing despair [23]. Adolescents and young adults who most strongly believe that it is acceptable to end one’s life are more likely to make a suicide plan than those who do not have such beliefs [24]. Longitudinal analyses of US data find that adolescents’ exposure to the suicide attempt of a friend or relative can trigger new suicidal thoughts and attempts [13, 25], particularly in girls [13], and after a friend’s suicide attempt [13], but that these effects fade with time [13]. They also identify a triggering effect of exposure to suicidal behaviour in a peer’s family member [26]. However, no British studies have described attitudes to suicide after suicide bereavement, despite this group featuring prominently in the suicide prevention strategies for England [7], Wales [8] and Northern Ireland [9]. Our objective was to explore the views of a large United Kingdom (UK) -based sample of young adults on whether their experiences of suicide bereavement had shaped their attitudes to suicide.

We conducted a national mixed methods cross-sectional study with the overarching objective of describing the impact of suicide bereavement on clinical and occupational outcomes in young adults. We chose to focus on young adults as an under-researched group, reflecting concerns about their vulnerabilities to suicide and social modelling of suicidal behaviour [27], their tendency to avoid accessing mental health services [28], and their priority status within UK suicide prevention strategies [6–9]. To access a large community sample of young adults, otherwise under-represented in health research, we chose to use the email systems of large higher education institutions, as we anticipated that this would elicit a better response than from a primary care mailshot. Previous analyses of quantitative data from this survey have tested specific hypotheses; finding an increased risk of suicide attempt and poor occupational functioning [3], and significantly higher stigma, shame, responsibility and guilt [29] scores in people bereaved by suicide compared with those bereaved by other sudden mortality causes. To develop our theoretical understanding of these associations we also collected free text responses to conduct an exploratory analysis of qualitative data in relation to a specific research question: How does a friend or relative’s suicide influence an individual’s own attitude towards dying by suicide?

## Methods

### Study design and participants

We invited all adults aged 18–40 (to include a young adult age range) who were working or studying at UK higher education institutions (HEIs) to participate in a closed, online study about sudden bereavement: the UCL Bereavement Study. The 18–40 age range was chosen to reflect the group of greatest policy interest at the time of designing the study [30]. We used a higher upper age limit than that used by the World Health Organisation (WHO) for young adults [27] in order to avoid collecting only the recent experiences of young bereaved adults.

We used the email systems of large institutions because we judged that this would be the best means of accessing hard-to-reach groups, whilst avoiding the biases associated with recruiting a help-seeking sample [31]. Our intention was that internet-mediated sampling would enhance recruitment of young men. We sampled from a diverse range of colleges and universities, including art, drama, veterinary and agricultural colleges; offering unique access to a large, varied but defined sample of young adults. We have described this sampling strategy previously [3].

All 164 HEIs in the UK in 2010 were invited to participate, and over 20% (37/164) agreed to take part, with a higher response (40%) from the Russell Group of universities (characterised by high income from research funding bodies). This provided a sampling frame of 659,572 staff and students. All participants were invited to take part in a survey of “the impact of sudden bereavement on young adults”; masking them to the study hypotheses. There was no accurate way of measuring response rate as the denominator of bereaved people was not ascertainable using routine data or survey methods. The majority of participating HEIs agreed to send an individual email invitation with embedded survey link to each staff and student member. For reasons of sensitivity (recent staff/student deaths) ten HEIs modified this strategy, for example by emailing students only, using their weekly news digest email, or advertising via staff and student intranet.

Inclusion criteria were as follows: people aged 18–40 who, since the age of ten, had experienced sudden bereavement of a close friend or relative. Early childhood bereavements were excluded to minimise recall bias and restrict our focus to adult cognitive processing of life events, using the age threshold for criminal responsibility in England and Wales. A close contact was defined as “a relative or friend who mattered to you, and from whom you were able to obtain support, either emotional or practical”. Sudden bereavement was operationalised as “a death that could not have been predicted at that time and which occurred suddenly or within a matter of days”. Exposure status was classified by responses to the question: “Since you were aged 10 have you experienced a sudden bereavement of someone close to you due to any of the following: a) sudden natural death (eg. cardiac arrest, epileptic seizure, stroke); b) sudden un-natural death (eg. road crash, murder or manslaughter, work accident); c) suicide?” Mode of death was defined subjectively by the respondent, and not by coroner’s verdict or death certificate, as perception of bereavement type was the exposure of interest.

### Procedures

Having consulted the COREQ guidelines on the design and reporting of qualitative research [32], an on-line questionnaire [3] was designed by AP, FS, DO, and MK. This was in consultation with a group of young bereaved adults and bereavement counsellors, who suggested which domains to cover and the appropriate wording of questions. It contained 119 fixed-response questions eliciting quantitative data on socio-demographic and clinical characteristics, and 20 open questions to probe specific dimensions of the impact of bereavement, eliciting free-text qualitative data. The questions were intended to be non-leading and neutral, so as to avoid assuming only negative outcomes of bereavement. The questionnaire was piloted as an open survey on the websites of the national voluntary sector organisations Samaritans, Cruse Bereavement Care, Survivors of Bereavement by Suicide, and Widowed by Suicide. Responses were reviewed and changes were made to the wording of specific questions, and branching was improved to reduce the time taken to complete.

Amongst the open questions, one probed respondents’ attitudes to their own death, and was worded: “*To what extent has their death made you fear that you may die in a similar way?”* This had no upper word limit. Respondents were invited to give as much or little detail as they wished, or to skip the question if it did not apply. Information provided to participants explained that the study was being conducted by a research team at UCL, including research psychiatrists (AP, DO, MK) and a medical sociologist (FS), and that data would be handled in accordance with data protection legislation.

All participants provided online informed consent. The study protocol was approved by the UCL Research Ethics Committee in 2010 (reference: 1975/002).

### Theoretical approach

In this study we took a primarily inductive approach, acknowledging an awareness of potentially conflicting theories in relation to the influence of suicide bereavement on attitudes to suicide. Joiner’s interpersonal theory of suicide proposes that exposure to the experience of a close contact’s suicide habituates an individual to death, reducing the associated fear [14]. This acquired capability for suicide could motivate that individual to enact underlying suicidal ideation [33]. The Integrated Motivational-Volitional (IMV) model of suicidal behaviour proposes that personal attitudes to suicide contribute to suicidal ideation, and social modelling of suicidal behaviour contributes to translating these thoughts into actions [14]. A personal awareness of the genetic basis for suicidality [34] might additionally engender beliefs about self-fulfilling prophecy, providing motivation for suicidal intent in the context of grief and distress. In contrast to these theories, it is possible that experience of the devastating impact on self and others might increase an individual’s motivation to avoid suicide [35]. Our exploratory approach acknowledged the possibility of these and other positions being evidenced in this dataset.

### Analytic approach

We used an adapted form of thematic analysis [36] to analyse free text responses to the question on attitudes to own death, restricting our analysis to respondents bereaved by suicide. Analysis was conducted collaboratively (by AP, HN, VA and NM) using NVivo software (QSR International Pty Ltd., 2014). After data familiarisation, responses were organised using a basic four-part classification: those who felt that, on balance, their experience of suicide bereavement had given rise to a fear of dying the same way, those who did not, those who gave uninterpretable responses, and those who stated “not applicable”.

We then moved to a more content-based, fine-grained approach, at which point we excluded brief responses such as “No”, “Yes”, “a little”, “somewhat”, or “to a large extent” as these provided few insights. Following discussions about emergent codes, the first 100 responses were coded independently by two members of the team (HN and VA) and compared for consistency of coding. Following assurance of a high level of agreement at this stage, regular discussions within the research team served to clarify the conceptual meanings of thematic codes as coding progressed. HN then coded the full dataset, building up a framework of new codes, sub-codes, collapsed codes, and higher-order categories in collaboration with AP, NM and VA. The revised framework was reviewed to explore both the distribution of responses across codes, and their meanings and content, leading to the emergence of further categories and higher-order themes. Data were then reviewed against higher-order themes as a final validation of the conceptual coherence of the analysis.

## Results

### Sample characteristics

**Fig. 1:**
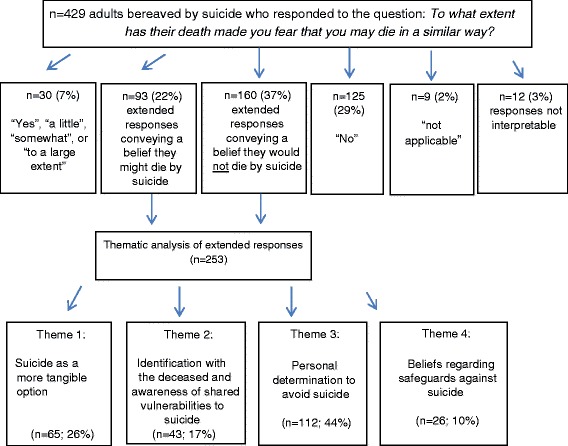
Participant flow and thematic framework

Of the estimated 659,572 bereaved and non-bereaved people receiving the email invitation, 5085 people responded to the questionnaire by clicking on the survey link, and 4630 (91%) consented to participate in the online study. Of the 3432 participants meeting inclusion criteria a total of 2408 (70%) provided free-text responses to the open question probing attitudes to own death. Within this group, 429 reported having been bereaved by suicide, 525 by sudden unnatural mortality causes, and 1454 by sudden natural mortality causes. We therefore analysed free text responses for a sample of 429 respondents (see Fig. 1 and Additional file 1: Table S1: Characteristics of study participants). This sample was predominantly female (82%), and of white ethnicity (92%). The mean age of respondents was 25.3, with a standard deviation of 5.9. The median time since bereavement by suicide was 4 years, with an inter-quartile range of 6.25. Roughly equal proportions reported the suicide of a family member (53%) versus that of a non-relative (45%). Among relatives, bereavement was by the suicide of a father (31%), cousin (18%), uncle/aunt (15%), brother (13%), mother (12%), grandparent (5%), sister (5%), or niece/nephew (1%). Overall, in 70% of cases the deceased had been male. The mean age of the deceased was 33.1 with a standard deviation of 15.7 years.

### Basic response characteristics

Of the 429 responses analysed to the question “To what extent has their death made you fear that you may die in a similar way?” a minority (see Fig. 1) were brief assenting responses such as “Yes”, “a little”, “somewhat”, or “to a large extent” (*n* = 30/429; 7%), or brief dissenting responses such as “No” (*n* = 125/429; 29%) or “not applicable” (*n* = 9/429; 2%). The remainder of responses (*n* = 265/429; 62%) contained more detail, varying in length up to 120 words, and typically consisting of three sentences. Responses conveying agreement with the question tended to be slightly longer in elaborating reasons.

Initial basic content-based coding of the 265 longer responses suggested that, after excluding those who indicated that the question was not applicable (*n* = 9/265) and those who gave uninterpretable responses conveying no clear attitudes to own death (*n* = 12/429), a smaller proportion of respondents (*n* = 93/265) felt that their close contact’s suicide had induced a fear of dying the same way than the proportion who felt that it had (*n* = 160/265). Combining brief and longer responses, whilst assuming that ‘not applicable’ represented a negative response, suggested that overall a third (*n* = 123/429; 29%) thought it conceivable that they might also die by suicide, but that two thirds (*n* = 294/429; 68%) did not feel this was likely. These proportions, however, mask complexity and expression of more mixed views, which are explored in more depth below.

### Themes identified

Our more in-depth analysis of extensive responses identified four key themes representing the attitudes that respondents expressed towards suicide following their experiences of suicide bereavement: 1) Suicide as a more tangible option; 2) Identification with the deceased and awareness of shared vulnerabilities to suicide; 3) Personal determination to avoid suicide; and 4) Beliefs regarding safeguards against suicide. In presenting our thematic analysis, quotes are given as provided in online typewritten responses. We corrected spelling errors only where due to omitted or inverted characters or where clarity was impeded. Percentages are given to denote the prevalence of differing views expressed, with the denominator as the sub-sample of 253 extended responses analysed. Some responses were coded in more than one thematic area.

#### Suicide as a more tangible option

About a quarter of respondents (*n* = 65; 26%) explained that the suicide of a close contact had made suicide seem more tangible. Having never previously considered it as an option, their experience of another’s suicide had rendered it a more realistic exit strategy from extreme distress, either for themselves or for others. For some within this group these thoughts provoked fear, but for others they did not.

*Normalisation of suicide*

Some participants felt that the suicide of a loved one had normalised suicide in a way that made it a less terrifying prospect, providing them with the reassurance that this might be a feasible escape and a means of controlling one’s destiny.

*“Knowing that if things get to be too difficult there's a way out”* (19 year old female, 9 years since uncle’s suicide)

*“If anything, it made it easier for me to consider and attempt suicide. If he had not done it, I probably would have not considered it in my lowest moments.”* (25 year old male, 9 years since brother’s suicide)

For a few participants bereaved by a friend’s suicide, the exposure had stimulated their curiosity in the experience.

*“I think about it every so often, gives me an urge to know what it feels like.”* (20 year old male, 3 years since friend’s suicide).

At the extremes, a small group of these respondents described their own suicide as inevitable, even to the point of romanticising it.

*“I feel almost certain that I will commit suicide one day. I think my body is too dramatic just to die naturally.”* (20 year old female, 4 years since friend’s suicide).

*Enhanced fear of suicide*

The suicide of a close contact had brought some a realisation that anyone in a state of extreme distress might be vulnerable to suicide, including themselves. This was sometimes articulated in terms of a loss of control.

*“After his death I didn't think anyone around me was capable of doing that, now I realise it can happen to anyone, especially after my attempt, I fear that I will end up dying in the same manner.”* (18 year old female, 1 year since friend’s suicide).

“*I've never had suicidal thoughts, but I have often had very anxious feelings of having been 'contaminated' by violence, of having had my boundaries of what is normal completely crashed …. it is essentially a fear that results from having 'seen too much' about how dark life can get and worrying that it will draw me in and take control of me”.* (31 year old female, 6 years since brother’s suicide).

For some this fear of suicide was transient and confined to the immediate aftermath of the bereavement, diminishing over time.

*“following [his] death it reared its ugly head that I too could do this ….. sometimes it still comes into my mind when I hear of a suicide but it is much less frightening than before as I know it is not a choice I want.”* (39 year old female, 10 years since brother’s suicide).

Alongside this new awareness of suicide as a personal threat, respondents expressed anxieties that others close to them might choose suicide. Those who had lost a family member tended to fear other family members dying by suicide, whilst those who had lost a friend tended to relate their fears to other friends. These fears had two dimensions: concern for the potential risk to friends or relatives, but also a dread of re-experiencing the same pain and grief.

*“I just worry about other people dying. I don't want to feel that level of pain and sadness again.”* (32 year old female, 15 years since friend’s suicide).

In cases where the suicide had been completely unexpected, with no apparent triggers, respondents re-conceptualised suicide as completely unpredictable. This created an anxiety that anyone in their social network was potentially at risk, again invoking a sense of loss of control.

*“Not at all. I am just worried about how easy my uncle could do this to himself. He seemed a self-controlled, financially secure, confident, loving, family man who enjoyed life. Finding no real reason is difficult. I now worry that it could happen to anyone in my life.”* (19 year old female, 8 months since uncle’s suicide).

This anxiety was particularly apparent where relative or friends were perceived to display vulnerabilities to suicide through mental ill-health or isolation.

#### Identification with the deceased and awareness of shared vulnerabilities to suicide

A minority (*n* = 43; 17%) of participants, predominantly relatives of the deceased, focussed on the role that mental illness had played in the suicide. They expressed fears about developing the same mental illness, and worsening to the point of becoming suicidal. Relatives were concerned about genetic vulnerabilities to mental illness and suicide, feeling powerless to escape inherited traits. These fears were particularly acute for those who had already experienced mental illness, and dreaded the prospect of losing control to depressive and suicidal thoughts.

*“After studying psychology at A Level I know her psychological disorder can be genetic and this scares me”* (21 year old female, 10 years since mother’s suicide).

*“To quite a serious extent. I have a very similar personality to my father with identical interests and similar character traits. I have experienced depression on a moderate scale on a handful of occasions and do fear that I have inherited a gene that would make me more prone to suicide. I worry that even if there is no biological inheritance other factors, for example being brought up with similar values and expectations, may make me more prone to suicide”* (23 year old male, 4 years since father’s suicide).

The minority who were non-relatives recognised shared personality traits and social difficulties, understanding these as contributing to suicide risk.

*“He killed himself because of the personal horrors he suffered. I suffered something similar, so sometimes wonder if I will become desperate too.”* (22 year old female, 6 years since friend’s suicide).

#### Personal determination to avoid suicide

Nearly half of respondents (*n* = 112; 44%) expressed a conviction, which for many had become stronger since their bereavement, that they would not die by suicide themselves. Their painful experiences of suicide bereavement had enhanced their awareness of the negative impact of suicide on family and close friends, creating a desire to avoid affecting others in the same way. Some in this group described an enhanced appreciation of life and a determination to live life to the full.

*“Her death has made me value life more and want to live life for as long as possible.”* (20 year old female, 2 years since friend’s suicide).

However others implied an underlying sense that suicide had become a more tangible option (Theme 1), representing an overlap between themes.

*“I will not commit suicide because I see what it can do to whole family circles. I don't think it's a selfish thing to do, sometimes people can't cope but it gives me strength.”* (21 year old female, 6 years since cousin’s suicide).

*“It's made me think that it's an option, but knowing what it does to those around you stops me doing it.”* (27 year old female, 7 years since friend’s suicide).

Some respondents described a new awareness of the violent nature of a suicidal death, recognising through their aversion to this that they lacked the capability to attempt suicide. Their responses conveyed a sense of taking control of their own destiny.

*“His suicide was quite violent … and if anything this strengthened the feeling that I would not want to, or be prepared to, die in the same way.”* (32 year old female, 2 years since friend’s suicide).

Some were struck by their own complete inability to understand the suicidal mind, even in someone known to them. This had reinforced their sense that suicide was not an option for them.

*“It has not. It made me realise that I am not really able to understand how you can be desperate enough not to think anymore that better days will come”* (30 year old female, 1 year since cousin’s suicide).

A few participants alluded to having re-evaluated their views on suicide, reaching the conclusion that this was now something that they would never choose. Again this overlapped with theme 1, in that these thoughts followed a realisation that suicide might be an option.

*“I made a decision in the months following his death. I sat all night and made the decision to keep living with all that entailed - old age, death by bus….cancer, murder, loneliness - so if ever my thoughts return to the way he died I remember that long night and the promise I am keeping every day of my life no matter how hard it is.”* (36 year old female, 10 years since partner’s suicide).

#### Beliefs regarding safeguards against suicide

A small minority (*n* = 26; 10%) of respondents felt that they needed to make active efforts to reduce the chances that they, or others, might become suicidal. These views were sometimes manifested in behavioural changes, as a means of controlling a presumed risk of suicide. An awareness of shared vulnerabilities to suicide (Theme 2) was implied as the basis for these behaviour strategies, representing an overlap in these themes, and again applying primarily to relatives of the person who had died by suicide.

Some described an awareness of the importance, for both themselves and others, of developing psychological coping strategies to prevent feelings escalating or becoming overwhelming. They mentioned an awareness of a need to avoid isolation, and to seek help at an early stage.

*“…I sometimes think about how I may get stuck into the same thought process later on in life but on the other hand I keep to a focus and know what feelings and thoughts to avoid as I have a point of reference with what happened to my father.”* (23 year old male, 2 years since father’s suicide).

*“Not at all- I know that if I ever felt sad, I could talk to my friends and brother and change my situation so that I felt happy again. If I ever felt depressed, I would ask for help before things got that bad because I know how suicide affects people close to you.”* (20 year old male, 5 years since father’s suicide).

*“Not really, but has made me more aware of making sure that anyone in a similar situation gets the help they need”* (22 year old male, 1 year since brother’s suicide).

In some cases a fear of dying a similar way had resulted in avoidant behaviour, conceptualised as a means of safeguarding themselves or others from suicide.

*“Their suicide was committed through an overdose of medication. For 5 years after the death I refused to take any medication fearing that I would accidentally take too much and end up ill.”* (20 year old female, 8 years since cousin’s suicide).

Avoidant behaviour strategies included blocking transmission of genetic vulnerabilities.

*“…I have a greater fear that [death by suicide] might happen if I have children, & this is one of the big reasons why I am terrified to have children - in case they are mentally unwell &/or die like that. Having watched what happened to my parents & my aunt & uncle, I could never do that.”* (26 year old female, 8 years since cousin’s suicide).

## Discussion

### Main findings

Our survey of young adults in British higher education institutions has identified a broad and complex range of attitudes to suicide following personal experience of a close contact’s suicide. The sense of gaining or losing control featured across all four of the themes that we identified. Respondents who described a determination to avoid suicide appeared to seek to exercise control over a perceived risk. Their awareness of the devastating and widespread grief the suicide had wreaked seemed to have deterred them from considering suicide themselves. Respondents who perceived their own susceptibility to suicide described a sense of inevitability, which they either battled against or submitted to. Among those who described an awareness of shared vulnerabilities to suicide, some appeared to convey a fear of being subject to a self-fulling prophecy, although this was not mentioned explicitly. This sense of inevitability was described by both friends and relatives, and was therefore not construed solely in terms of heritability. A small minority of our sample described a diminution of the fear of suicide, having re-evaluated it as a viable future means of escaping (and therefore controlling) threat. Such views corresponded to theoretical constructs of social modelling of suicidal behaviour, although again this was not mentioned explicitly. However, these views also demonstrated that terms such as ‘imitative suicidal behaviour’ convey an over-simplified passive process, rather than the more complex process of discovering meaning in a suicide and how such meaning influences one’s attitudes and behaviour, as detected in our data.

The ideas expressed in our study also map to theories of locus of control and research describing the association between an external locus of control orientation and suicidal behaviour [37]. The thoughts of inevitability and self-fulfilling prophecy expressed in these data are consistent with an external locus of control, while those describing resolutions to avoid suicide and behavioural changes are consistent with an internal locus of control. In keeping with sociological perspectives on bereavement, [38] our findings suggest that the specific affinity an individual has to a relative or close friend who dies by suicide (whether they were aware of shared vulnerabilities to mental health problems, or found the suicide completely unexpected) influences the meaning they assign to the suicide, and that this in turn influences their subsequent mental health and their attitudes to their own suicide.

Our two stage coding revealed the complexity and sometimes ambivalence of responses. The four themes identified in our fine-grained analysis did not map directly to the initial basic classification of those who did and did not fear dying by suicide. Rather than representing sub-groups of this binary classification, themes 1, 2 and 4 included the views of both those with a reduced fear of suicide and those with an enhanced fear. Only in theme 2, an awareness of shared vulnerabilities to suicide, were responses all contextualised in a fear of dying the same way. The degree to which young adults bereaved by suicide feared suicide appeared to be a function of how much they felt they could control their destiny, which influenced the strength of their determination not to die that way. Because of this complexity it is difficult to determine the degree to which a fear of suicide might be regarded as protective against suicide. Broadly our findings suggest that for some individuals the experience of suicide bereavement might increase their chances of modelling suicidal behaviour, whilst for others it might deter against suicide by triggering positive behaviour change. Whether or not an awareness of vulnerability translates into behaviour change is likely to depend on various personality traits, including an individual’s locus of control. Exploring whether someone bereaved by suicide feels in control of their own mortality is likely to be a useful means of uncovering their attitudes to their own suicide.

### Results in the context of other studies

Both quantitative and qualitative approaches have been used in the few studies attempting to explore attitudes to suicide after exposure to suicide. Some quantitative studies have used standardised attitudinal measures [39–41]. A population-based survey of 5117 Japanese adults bereaved by suicide compared with 2757 unexposed controls found that those bereaved by suicide were more likely to hold attitudes towards suicide as inevitable and not preventable [39]. This must be interpreted in the context of the greater tolerance of suicide in Japanese culture [42]. In a US study 85 young adults exposed to a peer suicide cluster in adolescence were more likely than 67 unexposed controls to normalise suicide but also more likely to find it incomprehensible [40]. Those who rated themselves as close to the deceased were more likely to identify with the attitudes “People often die by suicide on a whim”, and “If people want to die by suicide, we can’t stop them” [40]. A study of 54 workers in a US suicide intervention centre found that they had a greater fear of death than 62 psychology student controls, but such fears were not specific to suicide [41]. In an Australian study of 251 men who had recently attempted suicide, 67% described the feared impact on relatives as a deterrent against further suicide attempts [43].

These findings of normalising and permissive attitudes to suicide following exposure, a greater fear of death, and an awareness of the impact on others, are complex and highlight the need for qualitative approaches to gain a deeper understanding of underlying cognitions. Qualitative interviews with 36 suicidal young men in Northern Ireland showed that those exposed to peer suicide observed the devastating effects on friends and family, with the authors suggesting that this served as protective against suicide [35]. The informal accounts of US adolescents followed up after a friend’s suicide were that the experience had inhibited their own suicidal behaviour because of the perceived devastating effects on friends and family [44]. Our findings, within a large and representative dataset, of a deterrent effect, a normalising effect, and a fear of own suicide, are novel and demonstrate the complexity and diversity of attitudinal change after suicide. These findings build on the findings of the above studies, and provide much more detail as to how such attitudes had arisen and had subsequently been enacted in behaviour change.

### Strengths and limitations

To our knowledge this is the largest qualitative study of the attitudes of people bereaved by suicide towards their own risk of suicide. Effects of interviewer presence, including social desirability effects, were reduced through the anonymity afforded by an internet-mediated approach [31]. Our study did not use validated instruments to measure attitudes to suicide, but instead used open questions, allowing respondents an opportunity to elaborate their own views rather than respond to fixed-format questions. However, without interaction with respondents we were unable to probe the themes identified in more detail, or respond if participants became distressed. In qualitative terms, open-ended survey questions produce small amounts of data per person and are less able to access broad subjective experiences or life worlds than more personalised methods. In lacking a sense of respondents’ cultural frames, we were unable to explore how the meaning they ascribed to the suicide loss was influenced by their interactions with their social groups or societal culture. The wording of the question used to explore attitudes to suicide was guided by consultation with bereaved people for use in a sample bereaved by a range of natural and unnatural causes of sudden death. Use of the term ‘fear’ instead of ‘think’ was judged to be appropriate in this context, to avoid implying that such an outcome was inevitable. However, this may have encouraged people to include fear concepts in their responses, and made it harder to differentiate between responses alluding to probability of suicide versus fear of suicide. In analysing data from one question among a larger corpus of 20 qualitative questions there is a risk of losing context and failing to capture the totality of respondents’ positions. However, other questions addressed relationships, support experiences, and occupational impact, whereas this was the only one probing attitudes to suicide. Discussions between a team of four researchers enhanced reflexivity, and helped to ensure conceptual clarity and rigour in the analytic process. Our sampling of a large but defined population elicited a wide range of views, but our method resulted in over-representation of white individuals and highly-educated females. Given cultural dimensions to attitudes towards suicide, our findings may not be generalisable to other ethnic or social groups in the UK or beyond.

### Clinical and policy implications

Our work identified a sub-set of bereaved adults for whom suicide had become a realistic option, whether this was feared or not. Depending on other clinical factors, such as depressive cognitions, substance misuse, and personality traits, these individuals could potentially be at higher risk of suicide, driven by a fundamental change in attitudes since the loss. This group are of clinical concern given the associations between suicide bereavement and subsequent fatal [2] and non-fatal [3] suicide attempt. Psychiatric assessments routinely probe for family history of suicide as part of suicide risk assessment. Given recent epidemiological evidence [2, 3] they should also inquire about suicides in non-relatives. Our qualitative findings suggest that a history of bereavement by the suicide of a friend or relative should prompt an exploration of attitudes to suicide, perhaps using the question posed in this survey. The ethical challenge is whether psychosocial interventions with people contemplating suicide should invite consideration of the impact on close contacts, attempting to invoke this as a protective factor. Such work would need to be done skilfully to avoid worsening cognitive symptoms of guilt or anger. For those actively considering suicide it would be worth asking about methods considered, as clinical experience suggests that suicide-bereaved people describe enhanced awareness of a specific lethal method. Practitioners encountering anyone bereaved by suicide should direct them to available sources of support [45].

### Future research

The most valid means of investigating how attitudes to suicide are influenced by suicide bereavement is by measuring attitudes before and after exposure. However, as suicide is a relatively rare event this presents problems of study design. Longitudinal studies comparing problem-solving in individuals affected by significant life events such as job loss, bereavement, or relationship breakdown might identify cognitive markers of susceptibility to suicide attempt, informing the development of psychological interventions to mitigate this risk. Qualitative work with previously suicidal individuals is required to understand whether it is ethical and acceptable to discuss the impact of suicide on close contacts, and whether encouraging alternative coping strategies to suicide is feasible and acceptable to individuals who do not fear suicide. Recent English commissioning guidance on providing services for people bereaved by suicide describes the development of a range of support, including immediate outreach, group peer support, and individual counselling [46, 47]. This is in line with the perceived needs of this group [48]. However, whilst guidance is provided on service evaluation [49], there is also a need for randomised controlled trials of interventions designed to improve health and mortality outcomes, providing evidence that findings can be implemented in other settings.

## Conclusions

This qualitative analysis of British data from 429 adults bereaved by suicide reveals a range of attitudes to suicide, with a minority reappraising suicide as a realistic personal option. Others expressed a determination never to choose suicide, with some elaborating the positive steps they had taken to reduce their own and others’ risk. Given quantitative evidence of an increased risk of suicide in people bereaved by suicide, it is important to find ways of engaging and supporting this group. Professionals providing support after suicide bereavement should explore an individual’s attitudes to own suicide as part of suicide risk assessment and management. Sensitive handling of information divulged could help mitigate the theoretical risk of acting on suicidal thoughts, perhaps by exploring alternative coping strategies.

## Additional file

Additional file 1: Table S1. Characteristics of study participants. (DOCX 30 kb)

## Abbreviations

COREQ: Consolidated criteria for reporting qualitative research
HEIs: Higher Education Institutions
IMV Model: Integrated Motivational-Volitional Model
UCL: University College London
UK: United Kingdom
