# Supplementary material for: Attitudes to suicide following the suicide of a friend or relative: a qualitative study of the views of 429 young bereaved adults in the UK
Source: BMC Psychiatry. 2017 Dec 13;17:400. doi: 10.1186/s12888-017-1560-3 (PMC5729247; doi:10.1186/s12888-017-1560-3)
Supplement: Additional file 1: Table S1. — Characteristics of study participants. (DOCX 30 kb) [file 12888_2017_1560_MOESM1_ESM.docx]

|  |  |  |  |
| --- | --- | --- | --- |
|  |  | **Adults bereaved by suicide**  **(n = 429)** |  |
| **Demographic and bereavement characteristics** |  |  |  |
| **Gender** |  |  |  |
|  | **male *n (%)*** | 76 (18) |  |
|  | **female *n (%)*** | 353 (82) |  |
|  | **missing *n (%)*** | 0 (0) |  |
| **Age (years)** |  |  |  |
|  | **mean age (SD)** | 25.3 (5.9) |  |
|  | **missing *n (%)*** | 0 (0) |  |
| **Ethnicity** |  |  |  |
|  | **white *n (%)*** | 397 (93) |  |
|  | **other *n (%)*** | 32 (7) |  |
|  | **missing *n (%)*** | 0 (0) |  |
| **Relationship to the deceased** |  |  |  |
|  | **non-relative *n (%)*** | 193 (45) |  |
|  | **relative *n (%)*** | 229 (53) |  |
|  | **father** | 71 (31) |  |
|  | **cousin** | 42 (18) |  |
|  | **uncle/aunt** | 34 (15) |  |
|  | **mother** | 27 (12) |  |
|  | **brother** | 30 (13) |  |
|  | **grandparent** | 11 (5) |  |
|  | **sister** | 11 (5) |  |
|  | **niece/nephew** | 3 (1) |  |
|  | **missing *n (%)*** | 7 (2) |  |
| **Age when bereaved (years)** |  |  |  |
|  | **mean age (SD)** | 20.0 (5.5) |  |
|  | **missing *n (%)*** | 1 (<1) |  |
| **Gender of deceased** |  |  |  |
|  | **male *n (%)*** | 301 (70) |  |
|  | **female *n (%)*** | 123 (29) |  |
|  | **missing *n (%)*** | 5 (1) |  |
| **Age of deceased (years)** |  |  |  |
|  | **mean age (SD)** | 33.1 (15.7) |  |
|  | **missing *n (%)*** | 0 (0) |  |
| **Time since bereavement (years)** |  |  |  |
|  | **median (IQR)** | 4 (6.25) |  |
|  | **missing *n (%)*** | 1 (<1) |  |
|  |  |  |  |

### Supplementary Table 1: Characteristics of study participants

**Key:** SD=Standard Deviation; IQR=Inter Quartile Range
